# Primary care involvement in clinical research – prerequisites, motivators, and barriers: results from a study series

**DOI:** 10.1186/s13690-024-01272-x

**Published:** 2024-03-20

**Authors:** Julian Wangler, Michael Jansky

**Affiliations:** Centre for General Medicine and Geriatrics, University Medical Center of the Johannes Gutenberg, University Mainz, Am Pulverturm 13, Mainz, 55131 Germany

**Keywords:** Medical research, Health services research, Cluster-randomised controlled trial, Research network, Innovation Fund, General practitioner, Primary care

## Abstract

**Background:**

Long-term reinforcement in the role of primary care and improvement the healthcare system as a whole requires the involvement of GPs in clinical research processes. However, many clinical studies fail due to failure to achieve sample population targets amongst GPs and their patients. This issue has been identified and discussed, but effective strategies to overcome it are still lacking. One of the reasons is that the positions, requirements, and experiences of GPs on participating in clinical research have hardly been examined up to now.

**Methods:**

The years 2021 and 2022 saw three quantitative and qualitative surveys amongst GPs in Germany with the aim of shedding light on the attitudes, experiences, and potential issues regarding the involvement of primary care in clinical research projects and participation in cluster-randomised controlled trials (cRCTs) in a general sense. This overview summarises and abstracts conclusions gained from the exploratory series of studies and compares the results with the current research situation. From here, this contribution will then develop an approach towards optimising the integration of GPs into clinical research.

**Results:**

Most of the GPs asked associated clinical research with opportunities and potential such as closing gaps in healthcare, using evidence-based instruments, optimising diagnostic and therapeutic management, and reinforcement of multiprofessional healthcare. Even so, many GPs unsure as to how far primary care in particular would stand to benefit from studies of this type in the long term. Respondents were also divided on willingness to participate in clinical research. GPs having already participated in Innovation Fund projects generally saw a benefit regarding intervention and cost–benefit relationship. However, some also reported major hurdles and stress factors such as excessive documentation and enrolment requirements, greater interference in practice routines, and sometimes poor integration into project processes such as in communication and opportunities to play an active role in the project.

**Conclusions:**

Results from the studies presented provide indications as to how GPs perceive clinical research projects and cRCTs as a whole and from their existing project experience, and on the requirements that studies would have to meet for GPs to be willing to participate. In particular, making sure that clinical studies fully conform with GPs would play a major role; this especially applies to freedom to make medical decisions, limitation of documentation obligations, interference in regular practice routine, greater involvement in research planning, and long-term reinforcement in the role of primary care. Clinical research projects and cRCTs should be planned, designed, and communicated for clear and visible relevance to everyday primary care.

**Supplementary Information:**

The online version contains supplementary material available at 10.1186/s13690-024-01272-x.

**Table:** 

Text box 1. Contributions to the literature
• Many clinical studies fail due to failure to achieve sample population targets amongst GPs and their patients. This issue has been identified and discussed, but effective strategies to overcome it are still lacking. One of the reasons is that the positions, requirements, and experiences of GPs on participating in clinical research have hardly been examined up to now.
• In our results, most of the GPs asked associated clinical research with opportunities and potential such as closing gaps in healthcare, using evidence-based instruments, optimising diagnostic and therapeutic management, and reinforcement of multiprofessional healthcare. Even so, many GPs unsure as to how far primary care in particular would stand to benefit from studies of this type in the long term. Respondents were also divided on willingness to participate in clinical research. GPs having already participated in Innovation Fund projects generally saw a benefit regarding intervention and cost-benefit relationship.
• The studies presented have made a contribution to better understand how GPs perceive clinical research projects and cRCTs and under which prerequisites they are willing to participate in such activities. In particular, making sure that clinical studies fully conform with GPs would play a significant role; this especially applies to the medical decision-making freedom, limitation of documentation obligations, impediment to medical practice routine, greater involvement in research planning, and long-term reinforcement in the role of primary care. Clinical research projects and cRCTs should be planned, designed, and communicated for clear and visible relevance to everyday primary care.

## Background

Primary care plays an indispensable role in ensuring a functioning healthcare system. This applies to continuous (long-term) healthcare across the entire range of clinical conditions and complaints as well as patient types. However, it also applies to GPs in guiding their patients through the healthcare system by specifically referring them to other levels of care. Primary care participation in clinical research processes will play a central role in expanding primary care and other healthcare roles in a consistent and methodical fashion while also testing novel forms of healthcare and improving the healthcare system as a whole [1, 2]. New healthcare models – especially in the low-prevalence area – need to encompass sufficiently large patient cohorts for evidentially significant results, making primary care involvement inevitable in many cases [3–9].

However, GP-based interventions face significant hurdles in projects in clinical as well as healthcare research despite the significant role of primary care in clinical research and the potential benefits that may result. It is often a challenge to recruit a sufficient number of GPs for these studies, which usually involves a sophisticated cluster-randomised design in cRCTs [2, 10]. Various research projects and application areas have indeed shown recruitment of GPs to be a limiting factor in performing clinical projects involving primary care [1, 10–16]. Especially cRCTs usually require sufficiently sized study cohorts with failure to achieve patient recruitment goals often leading to insufficient statistical significance and even premature study termination [17, 18].

There are various reasons for insufficient overall recruitment and research participation amongst GPs [8, 13, 19]. Many studies have identified a lack of time and resources [11, 14, 15, 20, 21] and fear of administrative and documentation effort [21–23] as the main reasons for GPs to decide against participating in research projects. A lack of relationship with research and the problems this involves in understanding and implementing research methods have also been given as possible reasons [1, 3, 4, 6]. One qualitative study found GPs sometimes facing problems in enrolling patients in clinical research projects and supporting them throughout the intervention as they did not see themselves as being equipped with the comprehensive clinical research competence necessary [20].

Another factor is that Germany lacks a longstanding tradition of involving GPs in clinical research activities, unlike other countries [16, 24]. The healthcare and innovation systems may not be directly comparable, but studies in other Western countries have shown issues associated with recruiting and involving GPs in clinical research projects [5, 10, 13, 14, 16, 22, 23, 25–27]. As an example, only every third health study in primary care achieves its target patient cohort in Anglo-American countries as insufficient numbers of GPs can be recruited and/or too many leave the research projects early [5, 17, 28].

Summarising, recruiting GPs for major clinical healthcare studies is one of the greatest challenges facing healthcare research. This issue has already been identified and discussed, but there is still a lack of effective strategies towards overcoming it [2, 4, 6, 9, 10, 12, 16]. One of the reasons is that the positions, requirements, and experiences of GPs on participating in clinical research have hardly been examined up to now.

### Research interest and aim of study

Addressing the attitudes, experiences and potential issues involved in including primary care in clinical research projects and participation in cRCTs in a general sense crucially requires ascertaining the perspective of GPs.

The overview article summarises and abstracts the conclusions gained from an exploratory series of studies as well as the authors' own research experiences. The results are intended for comparison against the research situation up to now. This articles centres on the following issues:

- What attitudes do GPs have towards clinical research and its benefits for primary care?
- How far do GPs see barriers against participating in clinical research projects?
- Under what conditions would GPs be willing to participate in clinical research projects and cRCTs?
- What experiences have GPs had after participating in clinical research projects and cRCTs? Adding up the columns, what conclusions have they drawn from project participation?
- What would GPs like to see in the way of optimisation to increase the attractiveness of participation in clinical studies or cRCTs in the future?

In principle, the series of studies was about all types and forms of clinical research projects, i.e. not necessarily just about therapeutic interventions, but also, for example, about questions of quality of life, drug therapy and drug therapy safety, cross-sectoral care, medical guidelines and application adherence, geriatric care, telemedicine and eHealth/mHealth, delegation and substitution of services, care for vulnerable groups (e.g. family caregivers), communication with patients and promotion of health literacy, care in structurally weak or rural areas etc.

Overall, we aim to contribute to a better understanding of barriers and facilitators of the recruitment of GPs and their patients. With this in mind, we used the findings presented as a synopsis towards developing approaches towards optimising integration of GPs in clinical research.

## Methods

The studies included in this overall assessment include detailed surveys amongst GPs in Germany and in their willingness to participate clinical research activities and cRCTs as well as their experiences specifically in this regard. From the findings gathered together and presented in this contribution, we have drawn conclusions as to how clinical research projects might be designed towards making participation as attractive as possible amongst GPs.

### Study design and recruitment

**Fig. 1:**
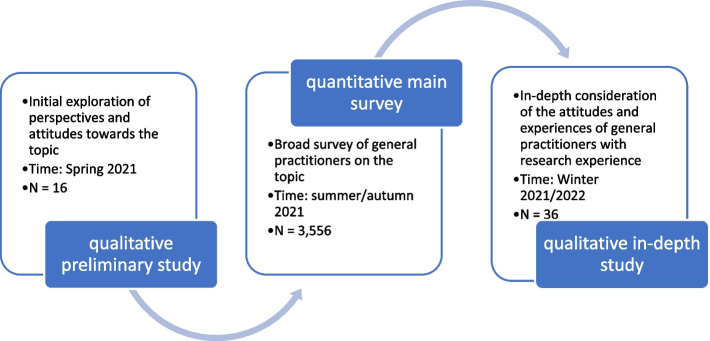
Order of the individual studies [29–31]

This analysis includes three surveys on German GPs posing a variety of questions with central areas of focus regarding participation in clinical research. All sub-studies were deliberately designed to be exploratory in nature, reflecting the paucity of research on this subject (see Fig. 1).

All 13,170 GPs with active practices in the federal states of Baden-Württemberg, Hesse, and Rhineland-Palatinate were invited to an online survey between July and November 2021 [30]; the survey was based on a smaller qualitative preliminary study that had already taken place [29]. This initial study served to collect general information on the topic in order to create the conditions for conducting a large quantitative study. The main study asked GPs for their attitudes, expectations on participation, and experiences from clinical research and especially the Innovation Fund, which serves as the central health policy instrument for promoting and financing new forms of healthcare in standard healthcare.^1^

A third study was qualitative in nature and functioned as an in-depth study, specifically aiming to capture the perspective of general practitioners with research experience. A total of 36 semi-standardised individual interviews with GPs already having participated in clinical and Innovation Fund projects were conducted between September 2021 and February 2022 [31] alongside the quantitative survey. Eleven regional physicians’ networks in the federal states of Rhineland-Palatinate, Hesse, North Rhine-Westphalia, and Schleswig–Holstein were involved in the recruitment process. This study mainly focused on investigating actual experiences amongst GPs from participating in research studies on the health services. With the help of the mentioned regional doctors’ networks, contact was established with a total of 36 GPs; interviews were conducted with all of them.

None of the studies included used any form of incentives.

### Development of survey instruments

Questionnaires and interview guidelines were developed for the quantitative and qualitative surveys on GPs as to their general participation and willingness to participate in clinical research and cRCTs; these questionnaires and guidelines took into account the authors’ previous research and recruitment experience in the Innovation Fund and evidence-based instruments [36–40] and general desk research (including Lech et al. [1] and Heytens et al. [34]). Both the quantitative main survey and the qualitative study contain the following main content areas: a) attitudes towards clinical research projects and their benefits; b) willingness to participate and corresponding prerequisites; c) experiences from taking part in specific projects; d) perceived optimisation potential.

The quantitative study contains a total of 25 questions. In addition to the standardized questions, which were often 4-point Likert scales, a series of open questions were used. The sociodemographic characteristics recorded were gender, age, practice environment, type of practice and patients per quarter. A pretest was carried out prior to data collection. For this purpose, the questionnaire was presented to 50 randomly selected GPs. The pretest showed that the questionnaire was easy to understand, structured and has complete answer categories.

The qualitative in-depth study included 20 questions. The focus was more on the experiences of GPs in clinical research projects. Here, too, a pretest was carried out in advance to check the comprehensibility and practicality of the guidelines.

### Data analysis

The SPSS 23.0 statistical package was used for evaluating the data from the quantitative survey studies. Apart from the descriptive analysis, Student’s *t*-test for independent samples was used to analyse for significant differences between the two groups. STROBE was used as the reporting statement for the main study.

Qualitative content analysis according to Mayring [41] was used as a basis for evaluating the qualitative interviews and open questions in the questionnaires. After transcription, we evaluated the interviews in a team using the MAXQDA software. In preparation, the written consultations were summarised with the essential information to gain an overview of the fundamental material. The text was then extracted in individual sentences or paragraphs depending on importance and expressiveness with units to be used in analysis previously determined (context, interview code, original text, paraphrasing, generalisation). The most important core statements were isolated, abstracted and summarised before forming categories. The categorical system created (see Multimedia Appendix 1) was based on the priorities set in the guidelines, repeatedly checked, and modified as necessary in the course of evaluation. We used the COREQ methodology as reporting statement for the qualitative study.

## Results

### Sample overview

The 3,556 fully completed questionnaires corresponding to a response rate of 27% were included in analysis [30]. Table 1 compares the sample obtained with reference data from the German Association of Statutory Health Insurance Physicians (KV) on the structure of GPs in Germany.

**Table 1:** Sample from the quantitative main survey [30] compared to reference statistics

	**Sample (*N* = 3,556)**	**Reference statistics**
Gender:	62% male, 38% female	58% male, 42% female^a^
Mean age:	55 (median: 55)	56 (median: 57)^a^
Practice setting:	51% town and city, 49% rural/small town	41% town and city, 59% rural/small town^a^
Type of practice:	61% individual practices, 32% group practices, 7% polyclinics or other establishments	56% individual practices, 38% group practices, 6% polyclinics or other establishments^b^
Patients per quarter:	25% 500–1,500, 28% 1,501–2,000, 47% > 2,000	Complete data unavailable
Membership in a physicians’ network	445	Complete data unavailable

^a^Based on health insurance research data in Rhineland-Palatinate (valid as of: 31 December 2020), available at: https://www.kv-rlp.de/institution/engagement/versorgungsforschung/

^b^Based on health insurance research data for Germany (valid as of: 31 December 2020), Available at: https://gesundheitsdaten.kbv.de/

The qualitative sample [31] comprised the following (see Table 2):

**Table 2:** Sociodemographic factors in the qualitative sample (*N* = 36) [31]

Type of office	18 joint offices, 18 single offices
Office environment	8 in small towns or rural communities, 15 in medium-sized towns, 13 in cities
Status	28 offices owned by the GP, 8 GPs in employment
Age	Ave. 52 years old
Gender	23 male, 13 female

### General results

Table 3 summarises the salient findings of the studies mentioned. These findings will be discussed alongside the research issues listed in the following.

**Table 3:**
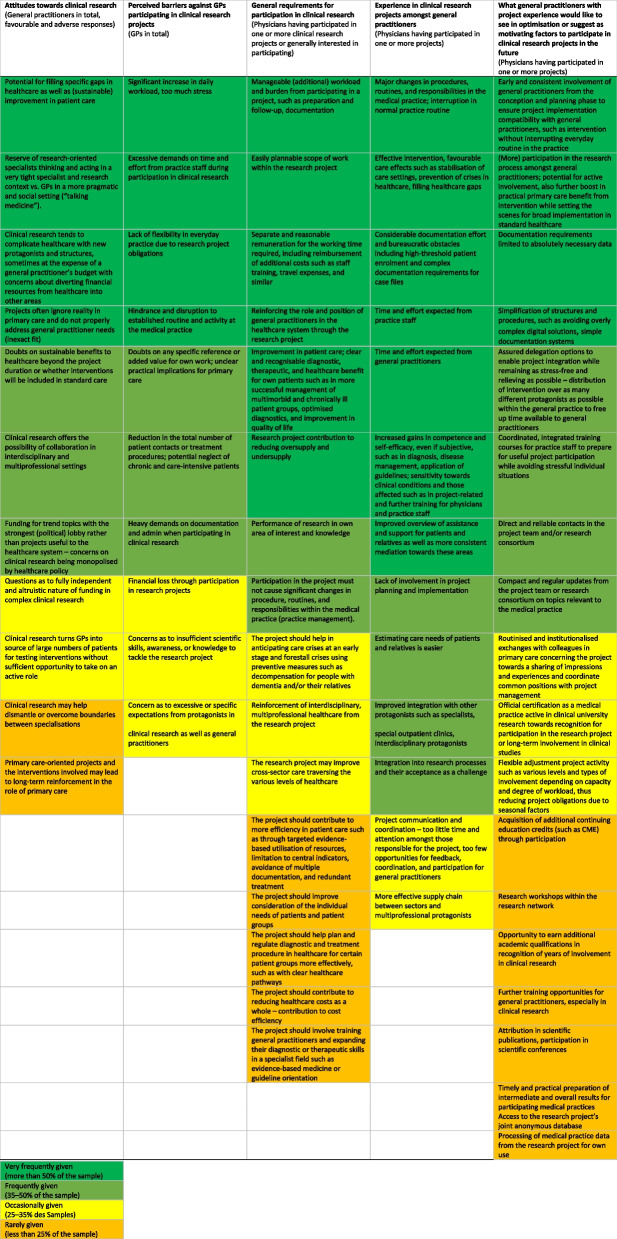
Attitudes, requirements, and experience amongst general practitioners towards clinical research projects and cluster-randomised controlled trials in the studies reviewed [29–31, 36, 37]

### Attitudes towards clinical research and healthcare benefit

Around half the GPs surveyed had an explicitly favourable attitude towards clinical research in all studies covered; the other physicians saw this rather negatively or did not take a clear position, which was mostly due to their stated unfamiliarity with scientific research [29–31]. Notably, the proportion of those reporting a favourable verdict in the quantitative study was significantly higher among urban than rural physicians (60% vs. 38%, *p* < 0.001). Around every third general practitioner associated clinical research with major benefit, while another third saw minor to moderate benefit [30].

Closer inspection reveals that a large proportion of those surveyed associated clinical research with considerable opportunities for the healthcare system, especially regarding identifying and closing gaps in care, using evidence-based instruments and procedures, and therefore optimising diagnostics and/or therapeutic management. Another benefit of clinical research projects according to respondents was their own contribution to reinforcing multiprofessional and cross-sectoral care, and therefore also the sequence of healthcare steps between the various medical and nursing protagonists.

“I do think it gives us an opportunity to benefit from targeted and sustainable improvements in taking care of our patients.” (I-8 m)

“Complex clinical research – Germany has long since been a bit of a developing country in getting general practitioners on board. This is where the vast majority of patients receive healthcare in everyday life. […] So, it’s definitely a step in the right direction.” (I-17f)

Even so, many GPs also doubted that clinical research would be an easy fit for the requirements of primary care, where pragmatic and social considerations (“talking medicine”) play a far greater role than a strictly research-based focus. Some therefore wondered how far primary care could benefit on a larger scale from involvement in this type of research project. Respondents especially mentioned addressing specific primary care needs and (sustainable) accuracy in interventions.

Apart from that, many respondents expressed concerns that clinical research could lead to issues in primary care in the long term with funds in the healthcare system being reallocated towards specialised structures even with the dependency of clinical research on primary care for studying larger patient cohorts and testing interventions. Some respondents during the interviews reported on their own experiences with projects involving new health protagonists such as special case managers with the concern that these new multiprofessional positions might ultimately come at the expense of primary care budgets and lead to “an over-engineered and bloated healthcare system” (I-24 m) [31].

“Clinical research encourages a kind of proliferation and chaos. New professional groups are constantly popping up, challenging the guiding role of general practitioners." (I-30 m)

With this in mind, the level of support from those surveyed was relatively low as to the prospect of clinical research projects and cRCTs leading to long-term reinforcement in the role of primary care. Physicians in urban areas anticipated this significantly more frequently than rural physicians (51% vs. 28%, *p* < 0.001) [30].

“The whole thing could also have a negative side. […] For example, I see a risk that these studies might ultimately bypass the reality of general practitioners too much and be of little use to us, or even a burden in the worst case.” (I-11 m)

"We’ve already seen that happen. GPs are recruited, but they’re more of a means to an end […] to feed study planners with patients.” (I-14 m)

Apart from that, a number of GPs expressed concerns that clinical research “does not necessarily support projects that the healthcare system needs;” rather, that it often focused on “politically selected topics and issues” (I-11 m). Some of the respondents also expressed doubts as to whether new healthcare models, such as those being tested in cRCTs, would ultimately find their way into standard care in practice [29, 31].

"Remember that these studies are subject to funding programmes lasting a few years. This is a high bar to overcome in successfully providing evidence of an intervention’s efficacy. I think many of these projects would just fizzle out for a whole variety of reasons." (I-25f)

### Perceived barriers to the participation of primary care in clinical research

Respondents saw the various aspects of additional workload as the greatest barriers facing general practitioner involvement in clinical research activities [29, 31]. This included increased amounts of work and significantly increased time and resource pressure for the entire practice team. The cost to flexibility in everyday practice due to research project commitments and intervention specifications was also seen as an issue.

"I’ve heard about this from a close colleague in general practice. He applied a clinically developed algorithm towards improving early diagnosis of liver disease. Sounds easy enough. But you can’t imagine the chaos that all the action guidelines caused in his medical practice. It sounded really awful.” (I-33m)

These perceptions are based on the fear of lasting detriment to established routine at the practice. Many respondents took the view that “general practitioners can’t afford to compromise on regular patient care for some special project especially in these times of high patient numbers and general practitioners in acutely short supply in some cases” (I-27f) [31]. A reduction in the total number of patient contacts and treatment programmes would therefore not be an acceptable condition for participation in research projects, according to many respondents.

“The thing is, you either join the project in full or not at all. That means either you’re willing to take on this added burden, or you’re not. But what if you want to contribute as a GP, but you can't get involved as much as the project requires in time or seasonality? Count me out. Because there’s no in-between in project participation, I mean as in flexibility." (I-29f)

This came with a high level of concern facing comprehensive and potentially escalating documentation and administrative obligations, such as in registering patients and filling out case files for the project. A few respondents also reported fearing substantial financial losses from participating in clinical research projects. Another key barrier was the lack of a research background amongst many GPs, so finding their way around the clinical procedure – especially in cRCTs – would mean a “transition and additional effort that shouldn’t be underestimated” (I-17f).

No fewer GPs saw a barrier in that those responsible for the project often failed to demonstrate any concrete benefit or added value for primary care from the intervention; practical implications for primary care remained unclear when recruiting from general practices for a study [31].

“Maybe it’s my lack of basic knowhow in research. But I'd like to know exactly what's in it for my patients and, of course, for me as a physician, before getting involved in something like this. I’m sure the project managers know what they have in mind, but they have a problem communicating it.” (I-11m)

### Willingness to participate, prerequisites, and reasons for participating in clinical research projects

According to the large-scale written survey of GPs, 31% of respondents were generally willing to consider participating in a clinical study or cRCT in the future, and another 24% reported that they had already participated in at least one associated study [30]. In contrast, 45% were fundamentally unwilling to participate in any clinical research project. Comparing age groups, 47% of physicians younger than the median age of 55 saw participation in a clinical research project would as an option vs. 20% of physicians aged 55 and over (*p* < 0.001).

In an open question, the respective physicians explained their willingness to participate as mainly due to curiosity and involvement in scientific research (35%), interest in or prior knowledge in the specific topic (35%), and a desire to help improve healthcare and quality of life for patients (45%).

Respondents not willing to participate explained their stance with consistently high workloads (54%), concerns about excess burden when participating in research activities (44%), and doubts as to the benefits of clinical scientific research in some cases (29%). Amongst GPs for whom taking part in clinical studies or cRCTs was out of the question, most doubted that these studies would find their way into standard healthcare (57%) or that they would be of any substantial benefit to primary care (58%).

Prerequisites played an important role for physicians responding that they would consider participating in a study or had already participated in one or more projects. Apart from likely diagnostic or therapeutic benefit for patients, they mainly focused on issues regarding the (limited) additional burden (such as preparation and follow-up, documentation, patient registration), appropriate remuneration, and structural improvement to the primary care setting. Respondents also saw importance in projects contributing to breaking down sector boundaries in the healthcare system and, above all, not interfering with normal operations and responsibilities in their medical practice [29–31]. Rural physicians in the quantitative survey emphasised the prerequisite that the project must not cause changes in practice routines far more often than city physicians (64% vs. 30% amongst city physicians, *p* < 0.001) [30].

“Committing yourself to studies like this isn’t trivial. They should see how they can accommodate general practitioners here. I think there’s still too little of that.” (I-6f)

### Experiences from taking part in specific projects

According to their own replies, 24% (875) of those respondents in the quantitative survey had already been involved or were currently participating in at least one clinical research project or cRCT [30]. The respondents comprised 92% urban and 8% rural physicians. Of the 875 respondents, 33% were individual and 67% group practices. Regarding age, 73% were younger than the mean, and 35% were networked with other physicians.

The information gained from surveyed reveals that most of the projects in which the physicians were participating or had participated focused on optimising a specific area of patient care, drug therapy or drug therapy safety, polypharmacy, extending regional and multiprofessional care networks, or promoting evidence-based medicine or compliance with guidelines. Many of the projects also involved telemedicine as well as enabling the delegation of care services. Projects focused on care in vulnerable groups such as caring relatives or people with disabilities or on promoting health and communication skills were less frequent.

However, some respondents emphasised that they had initially weighed up the feasibility of taking part in a large research study against their heavy workload [31].

“You have to think carefully about whether you can afford to take part in a study like this. You have to play it out in your head, even if things don’t turn out to be that serious.” (I-19m)

Two-thirds of respondents involved in the project reported that they needed to train members of the medical practice staff due to participation [30]. This especially applied to physicians participating in projects focused on drug therapy or specific medical conditions. Of the respondents, 80% reported severe (27%) or moderate (53%) complications vs. 20% with no complications as a result of participating in one or more research projects at their medical practice.

“That was an issue. The practice staff had to undergo a huge amount of preparation, the short-term training requirement was heavy… we were not informed about the type and extent of training from the start, and the training was scheduled at too short notice. This made normal office routine more difficult.” (I-26f)

The physicians involved in the project reported they were especially impressed by the results (from treatment) and optimised patient care (69%), improvement in cooperation with other care providers and sectors (52%), and enhancement of their diagnostic and therapeutic skills (40%) in response to an open question in the quantitative survey [30].

In contrast, physicians saw increased time pressure (66%) as well as considerable documentation requirements such as in registering patients and heavy paperwork in many cases (64%) as negatives alongside interference with practice routines and established procedures arising from project participation (55%) as well as too little involvement in research processes and decisions relating to the project for some of the physicians (43%). A few reported pressure from the project management to “recruit an unrealistically large patient cohort” (I-2 m).

“The hurdles and additional burdens shouldn’t be underestimated. I can understand why not all doctors can take part.” (I-25 m)

Some GPs complained that they did not have the research skills for rapid quick integration into the project or easy grasp of the procedure. On the other hand, some criticised the apparent lack of priority in bringing physicians up to speed on the research requirements such as in corresponding preparation courses [29, 31].

"Apart from that, we as general practitioners – especially in Germany – don’t have the academic background to keep up with these activities. This is a real problem that has to be addressed in medical studies in the long term if we really want to train general practitioners with an affinity for research.” (I-32f)

### Verdicts on project participation

GPs having participated in clinical studies or cRCTs draw a favourable overall conclusion in the general quantitative survey [30] on the benefits of the intervention tested. Of the respondents, 72% reported that care and treatment for the patients involved benefited very highly at 13% or rather highly at 45% vs. 18% less highly, 16% not at all, 8% difficult to say. Likewise, 66% rated the project participation benefit as clearly (43%) or slightly (23%) outweighing the effort involved vs. 11% about the same, 12% effort slightly outweighing benefit, 11% effort clearly outweighing benefit. Respondents rated projects covering healthcare in economically underdeveloped areas, drug therapy/safety, delegation and substitution, and cross-sector healthcare favourably for added value.

Of all the respondents having already participated in clinical trials or cRCTs, 15% reported prematurely ceasing participation. The main reasons they gave were excessive additional burden, (documentation) effort, and interference with practice routine. Some also mentioned inadequate opportunity for decision-making and participation. The qualitative interviews came to the same result [31].

“This project just got out of hand. They were constantly increasing the requirements for me as a physician without asking me. At some point, it became too much of a burden.” (I-31f)

Even so, most of the respondents stated that they would generally consider participating in other projects in the future provided the project promised worthwhile benefits for primary care from their point of view.

### Potential for optimisation

Physicians surveyed having taken part in clinical studies or cRCTs named several improvements they would like to see [30]. These involved strict limitations to documentation obligations (65%), a simple documentation system (62%), clearer organisation in project coordination (56%), making more flexibility possible in medical decision-making such as in calling in patients as well as decisions related to treatment, less severe interference with practice routines (49%), and reinforcement and improvement in structuring communication and cooperation between the physicians and other healthcare protagonists (37%). Finally, the physicians stated that they would appreciate (more) cost-based remuneration (34%).

The responses also demonstrate that the position of GPs should be reinforced further at various stages of a clinical study. Of all respondents, 57% saw importance in involving GPs more than before in study design and development. This would also include project-internal formats for structured participation such as research workshops as well as institutionalised exchange with colleagues and the research consortium.

“General practitioners simply just need to be more involved than before in designing and developing new studies and healthcare models. Once that happens, the studies will be more compatible with primary care and they’ll achieve their aims earlier." (I-23f)

GPs considered it particularly important for research projects to ensure the possibility of delegation allowing individual physicians to entrust practice staff members with project activities. Ensuring this across the board would save time and resources in the intervention. GPs also saw importance in integrated and coordinated training for the whole practice staff to prepare for workable project participation and avoid stressful individual situations. Flexible adjustment possibilities in project requirements such as varying levels and types of participation adjusted to capacity and degree of workload – such as reducing project obligations to account for seasonal factors – would also help prevent premature study cessation of GPs while also lowering barriers to entry [29–31].

GPs would appreciate more overall recognition of their commitment to clinical research. Some respondents suggested clinical practice status as recognition. A few respondents also raised the possibility of further academic titles as a result of years of involvement in clinical research.

## Discussion

### Principal findings and comparison with prior work

We have presented the main results from various surveys amongst GPs on their pervious experiences and future willingness to participate in clinical studies and cRCTs as a synopsis in the course of this contribution. These findings show GPs to be divided on whether to participate in studies of this type.

Overall, the attitudes of many GPs were notably favourable with regard to the fundamental benefits and added value from clinical research, and that opportunities for corresponding research projects were being taken such as towards identifying and closing healthcare gaps, intensifying application-oriented healthcare research, using evidence-based research instruments and procedures, optimising diagnostic and therapeutic management, and reinforcing multiprofessional healthcare. The research literature repeatedly described methodically embedding primary care into cross-sector, interdisciplinary structures as a major asset in clinical care models [15, 25, 36].

However, some GPs took a more critical and distanced attitude towards long-term goal orientation in corresponding studies. Some respondents were unsure as to what extent structures created by clinical-scientific care models could contribute in practice towards making the healthcare system more effective in the long term. Some expressed uncertainty as to whether primary care could actually benefit from such research participation in the long term.

Urban physicians in the quantitative survey sample [30] identified clear benefits from clinical studies and cRCTs, but their rural counterparts took a more cautious stance. This tallies with the general research findings that GPs in rural areas perceive lower added value in evidence-based structures and instruments [39, 40]. Likewise, most of the 24% of respondents having already taken part in cRCTs were located in urban areas with a greater variety of care services, which is often a prerequisite in effective clinical research [7, 9]. We did not find any significant gender differences in the studies we carried out; this contrasts with other individual studies on willingness of GPs to participate in research networks such as Virnau et al. [2, 42, 43]. Apart from the difference between urban and rural physicians, age is a factor that this study has in common: Openness to clinical research projects amongst the respondents decreased with age [2, 21].

Physicians fundamentally open to or already participating in research projects raised a number of requirements in this regard. Apart from added value for patient care, they emphasised manageable and plannable additional burden, impact on practice routines remaining tolerable, and structural reinforcement in the role of primary care. This tallies with results from previous surveys of primary care research networks (to be established) (see for example: [9, 24, 44–49]). A study by Güthlin et al. showed GPs to be especially interested in complex research projects if the topic seemed relevant to them and participation promised an actual benefit for the staff and patients of the practice. With this in mind, it hardly comes as a surprise that physicians having participated in clinical care models give especially favourable assessments of studies on topics such as rural care, drug therapy/safety, delegation, or sector cooperation. Other studies have also shown GPs to consider areas such as polypharmacy, drug safety and adverse drug effects, and multiprofessional cooperation models to be especially important [1, 2, 15, 29, 31]. Apart from that, many GPs currently would not want their medical practice just “researched,” but would rather help shape how these research projects are conducted [44, 45].

The conclusion reached by most of the GPs involved from participation in the corresponding studies is clearly favourable. This applies to healthcare and increase in treatment quality for the patients involved and to the cost–benefit relationship. Physicians found it easier to assess care needs of patients and their relatives, and recommend assistance services. Finally, there was a noticeable increase in subjective capability to perform diagnostics and disease management, and to apply the S3 guideline. Even so, some respondents described negative experiences and stress factors as reflected in documentation requirements and administrative effort, temporary but substantial changes in practice routine, deficits in project communication, and remuneration not matching the effort involved.

The results from the survey may be seen as confirmation of increased willingness amongst GPs to participate in empirical, evidence-oriented studies with the aim of optimising healthcare [15]. Especially younger GPs in urban catchment areas are increasingly basing their work on standardised, evidence-based interventions [39, 40]. Even so, a substantial proportion of general medical practices are fundamentally unwilling or remain reluctant regarding these research projects [1, 7–9]. This has resulted in a regional shortage of GPs available for recruitment in complex studies as reflected by project experience from the Innovation Fund in existence in Germany since 2015, often failing to achieve the original target cohorts of physicians and patients [50]. Lech et al. [1] provided one example in a contribution reporting on a cluster-randomised study to optimise outpatient dementia care. The authors reported difficulties in recruiting GPs despite using a wide range of recruitment strategies.

There is mounting evidence that combining these projects with everyday general practice care is not a smooth process, although the reasons for barriers and challenges to recruiting GPs for clinical research have hardly been investigated to date. This involves, on the one hand, immediate difficulties from an underlying shortage of time and resources in general practices [45]. GPs need to make the time required for project activities during consultation hours, which represents a major barrier to any research interest [43, 51]. This barrier may be raised further by the fear of a potentially escalating additional workload such as what GPs see as high-threshold registration of patients for the project, alongside documentation requirements and dealing with complex documentation systems such as digital case files. A low, tightly planned time investment for GPs and their staff always boosts the attractiveness of clinical research and may be of benefit to future recruitment [52]. On the other hand, GPs cast doubts on the match and fit of interventions in everyday primary care. This applies to project plans often conceived from a clinical-scientific perspective that then led to complications and limitations in primary care [44, 45, 49, 50, 53].

A review by Fletcher et al. [3] on GP-based clinical research identified barriers such as poor communication by study coordinators, difficulties experienced by GPs in understanding research methods, concerns about possible harm to patients, and the feeling of being overwhelmed by too many research requests without being perceived as genuine research partners. Routinised communication between all the stakeholders in every project phase plays an especially important role in enabling and improving practice-oriented research [54]. Apart from that, reliable and persistent contacts such as at university hospitals play a major part as an indispensable prerequisite for workable and cooperative relationships between resident GPs and clinical project management [55].

There are also indications that topics covered in clinical research projects do not always match the interests and perceived issues shared by gene, making it impossible to convince them to participate [2, 21, 43, 56–58]. This points to the need for continuous interaction between hospital-based primary care and GPs for continuous identification of everyday topics related to healthcare as relevant to GPs and their patients [59–62].

Beyond the issues already covered, Lech et al. [1] also discussed requirements for a specific recruitment of GPs. The contribution emphasised the benefit of greater concentration on (regional) physicians’ networks to specific recruitment in cRCT studies due to increased research interest, specific topic reference, and close coordination between the participating physicians [47, 63, 64]. A substantial proportion of the physicians involved in the studies were also members of a physicians’ network in the surveys presented [29–31, 36, 37].

Finally, consideration should be given to remuneration for participating GPs. GPs and their staff would welcome some financial reward for participating in clinical research even if most do not anticipate major financial losses from time spent in participation. One possibility would be increasing the remuneration amount with the number of patients enrolled into the study [65]. Apart from that, many GPs would benefit from reimbursement of additional expenses; this would help to ensure continuity and sustainability in clinical research networks [63]. Norway provides an example of good practice where physicians participating in research projects receive an annual fee for ongoing administrative work in addition to an hourly fee for each study participation [66].

### Most important takeaways from the studies presented

**Fig. 2:**
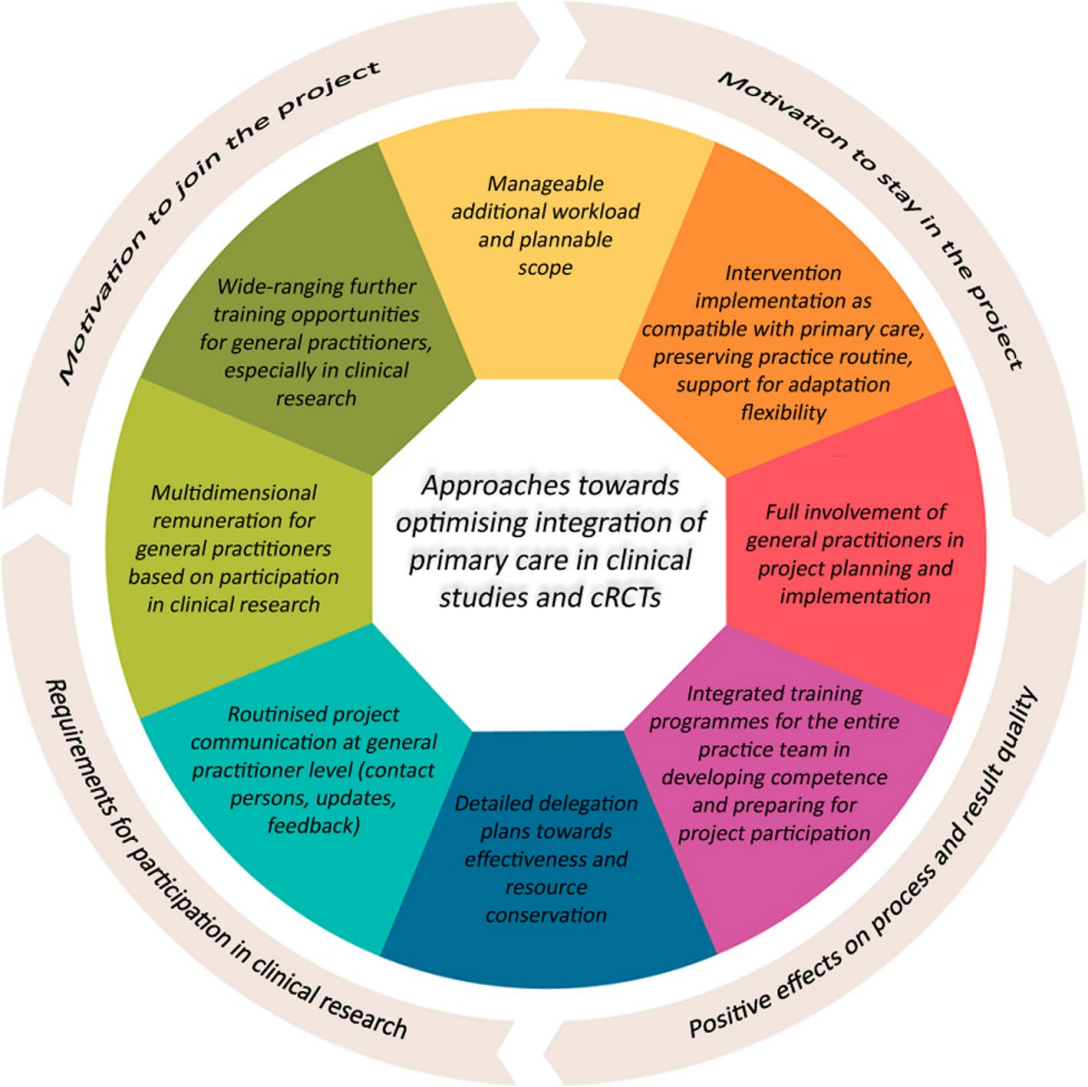
Approaches developed towards optimising integration of primary care in clinical studies (own diagram)

As shown, the findings obtained the from survey included in this contribution are largely consistent with existing research literature on primary care involvement in clinical research and cRCTs [55, 67, 68]. However, specific weightings and focal points in general practitioner positions as well as additional insights towards motivating GPs to take part in complex clinical research projects emerged during the studies. Figure 2 summarises the central takeaways.

GPs expressed a desire for a manageable and predictable additional workload such as in the complexity of the intervention to be used and in administrative and documentation tasks without excessively interfering with established practice routines [36, 39, 40, 49]. GPs also wished for more individual flexibility in action and decision-making extending beyond participation in research activities to involvement in evidence-based structures and instruments such as disease management programmes and guidelines. Examples of this included authorisation to take alternative approaches considered beforehand in these research projects or temporarily cutting down on project commitment without having to withdraw from the study entirely.

In addition, many GPs also expressed a strong desire for more involvement in shaping project activities and more inclusion in clearly structured communications during research projects. Many GPs advocate constant updates on research-related matters from project management, but also institutionalised, multi-layered exchange and feedback opportunities within the research network. GPs also found it important to use integrated and methodical training programmes and, wherever possible, detailed delegation plans for practice teams to demonstrate possibilities for implementing interventions while saving time and resources as far as possible. All this indicates that clinical research projects have still not always been compatible with the salient primary care setting up to now [16, 44, 53].

GPs saw it as desirable to approach rewards for participation in clinical studies not only in terms of remuneration alone, but also as a form of academic and research recognition. Some respondents saw a definite motivational factor in the possibility of receiving official certification as a university-associated research practice or a specific academic title in recognition of years of commitment to clinical research [44, 47].

Finally, training, and further training as a whole should undergo significant extension towards participation in research studies. The studies performed demonstrated that a sizable proportion of GPs were unsure about their research qualifications and current level of knowledge, leading to doubts as to their personal suitability for active research involvement. Germany has only seen increased efforts towards integrating research competence more firmly as a component of Medical Studies programmes in recent years [1, 37].

The main findings have demonstrated how it might be possible to recruit more GPs in the future. In the opinion of the authors, consistent implementation of these resulting clusters will not only exert a favourable effect on motivation to join but also to remain in the project while also improving process and result quality in cRCT studies. This would also create more favourable general conditions for GPs to take an active part in clinical research in the future. It would also be important to align research projects with topics that GPs see as relevant for these optimisation approaches to materialise, and also to convey the specific benefit and added value for primary care in a clear fashion when addressing physicians [2].

### Strengths and limitations

The studies presented in this contribution are to the best of our knowledge amongst the few empirical studies that have been published so far with an in-depth focus on attitudes, acceptance, expectations, and experiences of GPs towards participation in clinical research projects. However, the study cannot make any representative claims in the strict sense due to the limited number of cases and regional recruiting focus. We must also take into account that the focus of the surveys was also largely placed within the Innovation Fund context. Particularly extensive, complex, and also cost-intensive clinical research projects in Germany are financed by the Innovation Fund, which is not necessarily representative of any other type of clinical research.

In addition, the proportion of GPs involved in research is noticeably overrepresented in the quantitative survey sample compared to the total number of GPs, so selection bias needs to be considered. This implies that the survey addressed physicians with a greater interest or commitment to the topic at hand in contrast to physicians with no connection to clinical research, who have presumably participated to a lesser extent in clinical research. The responses from respondents on the main topics of respective research projects should also be seen within this context. The ranking order of responses listed reflects the topical interest of GPs, but the number of projects available for the respective subject areas may cause a bias in this information.

Even so, the heterogeneous random sample taken approximated to the general population of GPs in important aspects (see Table 1). The exploratory approach combining quantitative and qualitative components allowed a wide range of general practitioner perspectives, attitudes, and experiences to be documented.

The present study has not looked into how far the projects the responding GPs took part in were implemented, co-managed, or coordinated by primary care institutes. These institutes have gathered a wealth of experience in research collaboration with GPs. Future studies should therefore focus on whether the study conditions for GPs could be more favourable in cooperation with primary care institutes.

The present studies have also left aside the extent to which settings other than clinical studies may be more suited to primary care regarding willingness to become involved in scientific research. Studies from primary care suggest that the research practice model may potentially achieve more effective recruitment and participation [44, 47]. In this respect, results from the present study may be compared with results from a survey to be suggested here documenting the experiences of GPs specifically in the research practice setting. This type of survey would be feasible on a larger scale in view of the recent emergence of larger research networks coordinated by primary care institutes.

## Conclusions

Results from the studies presented provide indications as to how GPs perceive clinical research projects and cRCTs as a whole and from their existing project experience, and on the requirements that studies would have to meet for GPs to be willing to participate. Future research projects on primary care-based interventions should redouble their efforts at reflecting the positions, needs, and experiences of GPs. This would enable us to even out the hurdles and challenges perceived by GPs in participating in projects of this type. In particular, making sure that clinical studies fully conform with GPs would play a significant role; this especially applies to the medical decision-making freedom, limitation of documentation obligations, impediment to medical practice routine, greater involvement in research planning, and long-term reinforcement in the role of primary care. Clinical research projects and cRCTs should be planned, designed, and communicated for clear and visible relevance to everyday primary care.

### Supplementary Information

**Additional file 1: Multimedia Appendix 1.** Categorical system, qualitative in-depth study.

## Data Availability

All major data generated or analyzed during this study are included in this published article. Additional information can be provided on request made to the corresponding author.

## Footnotes

1 The year 2015 saw the Innovation Fund established as part of the Federal Joint Committee (G-BA) [32]. As a health policy instrument, the aim of the fund is to promote evidence-based development in pay-as-you-go healthcare by developing new healthcare concepts [32–35]. An annual funding volume of €200 million has been secured for the project in the current funding phase with funding provided by the statutory health insurance companies and health fund.
