# Supplementary material for: Primary care involvement in clinical research – prerequisites, motivators, and barriers: results from a study series
Source: Arch Public Health. 2024 Mar 20;82:41. doi: 10.1186/s13690-024-01272-x (PMC10953082; doi:10.1186/s13690-024-01272-x)
Supplement: Supplementary file 1 — Additional file 1: Multimedia Appendix 1. Categorical system, qualitative in-depth study. [file 13690_2024_1272_MOESM1_ESM.docx]

**Appendix 1: Categorical system**, **qualitative in-depth study**

**1) Attitudes towards clinical research projects and perception of their benefits:**

a) Principal benefit for the improvement of (regular) care

b) Neutral research funding vs. political influence

c) Application-oriented or sustainable optimization of incorrect or incomplete care

d) Primary care physician participation in clinical research

e) Addressing of primary care needs and the accuracy of interventions

f) Position of general practitioners in the context of clinical research

g) Structural changes of healthcare due to clinical research interventions

**2) Willingness to participate and its requirements:**

a) Interest in or participation in patient-related and clinical research

b) Optimization of patient care and quality of life as well as diagnostic and/or therapeutic benefits

c) Effort and burden

d) Remuneration

e) (Structural) revaluation of primary care work

f) Changes in work processes, practice routines and responsibilities

g) Improving cross-sector and multi-professional care

h) Training or development of diagnostic or therapeutic skills

i) Optimization of structuring and efficiency of patient care

j) Better anticipation of care crises

**3) Experiences in participating in specific projects:**

a) Type of recruitment or enrollment

b) Training needs with regard to the practice staff

c) Changes or restrictions in practice operations due to project participation

d) Experiences and observations with regard to the benefit of the intervention

e) Effort-benefit ratio of project participation

f) Assessment of individual elements of project participation

g) Complete termination of project participation or premature termination

h) Willingness to participate in future projects against the backdrop of experiences made

**4) Perceived optimization potential:**

a) Limiting administrative effort

b) Organizational structuring and project coordination

c) Enabling GP decision-making flexibility

d) Limitation of interventions in practice processes

e) Management of interdisciplinary communication

f) Effort-based remuneration

g) (More) involvement of general practitioners in project design and implementation
